# Skin Mast Cells Contribute to *Sporothrix schenckii* Infection

**DOI:** 10.3389/fimmu.2020.00469

**Published:** 2020-03-19

**Authors:** Qingqing Jiao, Ying Luo, Jörg Scheffel, Peng Geng, Yuhan Wang, Stefan Frischbutter, Ruoyu Li, Marcus Maurer, Zuotao Zhao

**Affiliations:** ^1^Department of Dermatology, First Hospital, Peking University, Beijing, China; ^2^Department of Dermatology and Allergy, Charite–Universitätsmeidzin Berlin, Berlin, Germany; ^3^Department of Dermatology, The First Affiliated Hospital of Soochow University, Suzhou, China; ^4^National Clinical Research Center for Skin and Immune Diseases, Department of Dermatology, Peking University First Hospital, Beijing, China

**Keywords:** *Sporothrix schenckii*, sporotrichosis, skin, mast cells, TNF, IL-6

## Abstract

**Background:** *Sporothrix schenckii* (*S. schenckii*), a dimorphic fungus, causes sporotrichosis. Mast cells (MCs) have been described to be involved in skin fungal infections. The role of MCs in cutaneous sporotrichosis remains largely unknown.

**Objectives:** To characterize the role and relevance of MCs in cutaneous sporotrichosis.

**Methods:** We analyzed cutaneous sporotrichosis in wild-type (WT) mice and two different MC-deficient strains. *In vitro*, MCs were assessed for *S. schenckii*-induced cytokine production and degranulation after incubation with *S. schenckii*. We also explored the role of MCs in human cutaneous sporotrichosis.

**Results:** WT mice developed markedly larger skin lesions than MC-deficient mice (> 1.5 fold) after infection with *S. schenckii*, with significantly increased fungal burden. *S. schenckii* induced the release of tumor necrosis factor alpha (TNF), interleukin (IL)-6, IL-10, and IL-1β by MCs, but not degranulation. *S. schenckii* induced larger skin lesions and higher release of IL-6 and TNF by MCs as compared to the less virulent *S. albicans*. In patients with sporotrichosis, TNF and IL-6 were increased in skin lesions, and markedly elevated levels in the serum were linked to disease activity.

**Conclusions:** These findings suggest that cutaneous MCs contribute to skin sporotrichosis by releasing cytokines such as TNF and IL-6.

## What Is Already Known About This Topic?

- Sporotrichosis is an infection caused by *Sporothrix schenckii* (*S. schenckii*) and can be particularly harmful in immunocompromised patients.
- In addition to their critical role in allergic disorders, Mast cells (MCs) have been recognized for their complex role in fungal infections.

## What Does This Study Add?

- Wild type (WT) mice that are infected with *S. schenckii* develop larger sporotrichosis lesions than MC-deficient mice. This is associated with the production of the proinflammatory cytokines IL-6 and TNF by MCs.
- The severity of *S. schenckii* infections in humans correlates with IL-6 and TNF levels.
- MCs contribute to the progress of skin sporotrichosis.

## What Is the Translational Message?

Mast cells and mast cell-derived cytokines should be further explored for their role in sporotrichosis.

## Introduction

Sporotrichosis is a subacute or chronic infection caused by the dimorphic fungus *Sporothrix schenckii* (*S. schenckii*), which has a worldwide distribution (1–3). During the last 10 years, the overall incidence of sporotrichosis has continued to increase, especially in the northeast of China. In humans, this mycosis mainly affects the skin of infected patients. Although less common, other organs may also be affected, such as the lungs, joints, bones, and even the brain, especially in immunocompromised individuals. Treatment usually involves the use of antifungal agents and/or surgical excision. However, patients are frequently resistant to treatment, there have been reports of relapse or progression during therapy, and problems due to a lack of tolerability of antifungal drugs occur often (4). Thus, research is necessary to identify and characterize the underlying mechanisms of *Sporothrix* infection, which may lead to the development of new and better therapeutic options.

Mast cells (MCs) are strategically located in tissues at the interface with the environment such as the lung, the gut, and the skin. Therefore, MCs are perfectly positioned to orchestrate defense mechanisms for invading pathogens including fungi (5, 6). Our recent review of the literature on the role of MCs during innate immune responses against invading fungi revealed that MCs function as positive or negative immunoregulatory cells depending on the situation (7). Examples of beneficial host responses against fungal infections by MCs include their effects in promoting the restriction of infections or the resolution of inflammation (8–12). Negative MC responses to fungal infections include the promotion of a wider dissemination of antigens, increasing the severity of the infection and inflammation, and promoting the development of atopic disease (13–25). These findings challenge and extend the current notion of MCs primarily serving the host rather than the pathogen when immune responses are raised at sites of infections.

As of now, there is only limited information on the contribution of MCs to immune responses against *S. schenckii* infection (19, 22). Using an experimental mouse model of systemic *S. schenckii* infection, Romo-Lozano et al. found that mice without functional peritoneal MCs presented with a decreased fungal load in organs and a reduced severity of clinical manifestations (26). This suggests that MCs may promote the dissemination of *S. schenckii* and increase the severity of the infection. However, the major clinical manifestations in most cases of sporotrichosis occur in the skin, after traumatic inoculation or zoonotic transmission, and infections with *S. schenckii* primarily results in cutaneous sporotrichosis (fixed cutaneous, lymphocutaneous, or disseminated forms) (27). As of now, the role of MCs in sporotrichosis of the skin has not been investigated and, thus, remains to be characterized and defined (28–31). Consequently, the purpose of the current study was to investigate the *in vitro* response of skin MCs to *S. schenckii* involvement in a murine model of experimental cutaneous sporotrichosis, and their role in human skin infections with *Sporothrix*.

## Materials and Methods

### Mice

All mice used in the experiments were on a C57BL/6 background. Wild-type (WT) C57BL/6-*Kit*^+/+^ and MC-deficient C57BL/6-*Kit*^*W*^*/Kit*^*W*−*v*^ mice were bred and housed at our facilities, and wild-type (WT) Cpa3-Cre/Mcl-1^+/+^ and MC-deficient Cpa3-Cre/Mcl-1^fl/fl^ (Hello Kitty, HK) mice were also obtained from breeding colonies of the animal facilities of the Charite - Universitätsmedizin Berlin. All mice were kept under specific pathogen-free conditions, and all experiments were conducted according to institutional regulations.

### Fungal Strains

The *S. schenckii* wild-type strain M-64 (ATCC MYA 4822) was a donation from Prof. Sandro Rogerio de Almeida's laboratory (Department of Clinical and Toxicological Analyses, School of Pharmaceutical Sciences, University of São Paulo, SP, Brazil). The *Sporothrix albicans* wild-type strain (*S. albicans*, ATCC® 201162^TM^) was purchased from ATCC. Initially, the isolates were subcultured from conidia until complete differentiation into the yeast form. Yeast cells were grown in brain-heart infusion broth (BHI, HB8297-1, Hopebio, China) for 7 days at 37°C with constant rotary shaking at 150 cycles/min, in order to yield a high percentage of yeast conversion. Then, yeasts were harvested from the BHI culture by centrifugation, washed twice and adjusted to the required concentration in sterile phosphate buffered saline (PBS), pH 7.4, and stored at 2–8°C until use.

### Murine Model of Skin Sporotrichosis

To study the role of MCs in skin infections with *Sporothrix*, we infected MC-deficient mice (HK) and WT littermates (C57BL/6) with *S. schenckii* subcutaneously by injecting 30 μl of PBS containing 3 × 10^7^ cells of *S. schenckii* yeast into the right foot pad, and PBS vehicle into the left foot pad as autologous control. Mice were then examined every 3 days during 5 weeks by evaluating the thickness, width, and length of foot inflammation with an electronic micrometer and the presence of skin ulceration or scarring in foot pad by photographing. The lesion volume was calculated (in mm^3^) as ellipsoids [(a/2×b/2×c/2)×4/3×π].

### Determination of Fungal Burden

The fungal burden of infected skin sites of mice was measured by counting colony-forming units (CFU). Briefly, the organs were separated, weighed, and homogenized in sterile PBS with a tissue grinder. Samples (100 μL) of each homogenate were seeded on Petri dishes containing BHI agar and incubated at 37°C. The material was incubated for 7 days at room temperature, and the number of colonies formed on each plate (CFU) was counted, and the mean of viable fungi was then calculated for each group. The results were expressed as CFU/g tissue.

### Histology

Tissue samples from infected mice were fixed in 10% formalin for 16 h, dehydrated in alcohol, and embedded in paraffin. Histological analyses of foot, spleen, lungs, liver, and lymph node were performed with a Zeiss Axioplan 2 Imaging microscope using a Zeiss AxioCam camera run by AxioVision Rel. 4.8 software. Giemsa stain was used to identify degranulated MCs.

For immunohistochemistry staining, sections were deparaffinized in xylene for 10 min and then rehydrated in graded alcohols and water. TNF staining was performed according to the manufacturer's instructions (DakoEnVision^+^ System- HRP Labeled PolymerAnti-Rabbit). Briefly, antigen retrieval was executed in citrate buffer (pH 6.0) in 95°C for 30 min. After blocking of non-specific binding with Block Dako for 10 min at room temperature, sections were incubated with primary antibodies against human and mouse TNF (ab9739, Abcam) in a humid chamber at 4°C overnight. Then sections were treated with H_2_O_2_ for 5 min at room temperature (RT). After three washes with TBS, sections were incubated with polymer secondary anti-rabbit secondary antibodies for 30 min, washed three times in TBS, and incubated with substrate AEC for 5 min. Slides were rinsed in TBS and counterstained with Mayer's hematoxylin (ab128990, Abcam; negative control = omission of the primary antibody). For IL-6 staining (Dako REAL™ Detection System, Alkaline Phosphatase/RED, Rabbit/Mouse, K5005), antigen retrieval was executed in citrate buffer (pH 6.0) in 95°Cfor 30 min. After blocking with avidin block, biotin block and 5% goat serum for 15 min each at room temperature, sections were incubated with primary antibodies against mouse IL-6 (sc-1265, Santa Cruz Biotechnology) and human IL-6 (sc-130326, Santa Cruz Biotechnology) in a humid chamber at 4°C overnight. After three washes with TBS, sections were incubated with biotinylated goat anti-rabbit secondary antibodies for 30 min, washed three times in TBS and incubated with ABC and substrate. Slides were rinsed in TBS and counterstained with Mayer's hematoxylin as described before. The expression of TNF and IL-6 was assessed by histomorphometry (Zeiss Axioplan 2 Imaging microscope using Zeiss AxioCam camera run by AxioVision Rel. 4.8 software).

### BMCMC Cultures and Reconstitution of Mast Cell-Deficient Kit^W^/Kit^W-v^ Mice

Femoral and tibial bone marrow from 4 to 6-week old C57BL/6 mice was cultured in DMEM plus recombinant stem cell factor (SCF, 20 ng/mL, Biolegend) and recombinant interleukin-3 (IL-3, 20 ng/mL, Biolegend) containing medium for 4–8 weeks to generate populations of bone marrow-derived cultured mast cells (BMCMCs) that were >95% pure as assessed by flow cytometry counting CD117 and FceRI double positive cells.

BMCMCs (10^6^ in 50 μl 0.9% NaCl) were injected intradermally and mice were used for experiments, together with sex- and age-matched MC-deficient Kit^W^/Kit^W−v^ and Kit^+^/^+^ mice, 4 wk after adoptive transfer. Reconstitution of cutaneous MC populations was confirmed by histomorphometric analyses of paraffin-embedded, Giemsa-stained sections of injected skin (Supplementary Figure 8).

Assessment of degranulation by flow cytometry, Wild-type BMCMCs were incubated with different concentrations of *S. schenkii* yeasts, medium alone as a negative control, or PMA/ionomycin as a positive control, respectively. After 0.5 h of incubation with *S. schenkii* yeasts, BMCMCs were stained with APC anti-mouse CD63 antibody (143906, Biolegend). APC Rat IgG2a, κ- antibody (400512, Biolegend) was used as isotype control. Cells were analyzed with a Coulter Epics XL Flow cytometer or a Coulter FC 500 ANALYZER (Beckman Coulter). The relevant data were obtained and analyzed using FlowJo software, version 7.6.

### Cytokine Release Assay

BMCMCs (5 × 10^6^) were incubated with or without yeasts (9.4 × 10^7^, 4.7 × 10^7^, or 2.35 × 10^7^) of *S. schenkii* or *S. albicans* at 37°C in supplemented RPMI 1640 medium under an atmosphere of 5% CO_2_ for 24 h. Cell-free supernatants were collected and stored at −80°C until assayed for TNF and IL-6. Then, we used ELISA kits (Biolegend) to determine the levels of TNF, IL-6, IL-1β, IL-10, and MCP-1 in cell culture media according to the manufacturer's instructions. Serum levels of TNF and IL-6 in sporotrichosis patients were determined using human TNF or IL-6 enzyme-linked immunosorbent assay kits, respectively (R&D Systems). The results were expressed in pg/ml.

### Patients and Control Subjects

Ethical approval from the Ethics Committee of Department of Dermatology, First Hospital, Peking University (Beijing, China) was obtained prior to the study. A total of 15 sporotrichosis patients in Han Chinese population (*n* = 15, male: 6, female: 9; aged 30–50 years, *n* = 4, mean 43.75; aged 50–70 years, *n* = 7, mean 61;aged 70-90 years, *n* = 4, mean 78;) were included after informed consent (Table 1). The definitive diagnosis of sporotrichosis was established by isolation of the fungus from skin specimens (exudate, scales, and skin biopsy), which were sent to the Laboratory of Mycology. The definitive diagnosis was made by isolation of *Sprothrix* species as previously described. The control group consisted of 15 plastic surgery-derived skin samples of healthy individuals.

**Table 1:** Clinical data for the 15 sporotrichosis cases in the present study.

**Patient no**.	**Clinical form**	**Disease duration (month)**	**Location**	**TNF (pg/ml)**	**IL-6 (pg/ml)**
1	Fixed	2	Face	21.34044297	21.44581527
2	Lymphocutaneous	1	Lower limbs	71.20012408	72.77879742
3	disseminated cutaneous	4	Lower limbs	86.87250613	94.53421187
4	Fixed	12	Upper limbs	27.51110135	24.09024543
5	Fixed	1	Lower limbs	34.27076872	35.81438507
6	Lymphocutaneous	3	Lower limbs	54.74241527	84.46412093
7	disseminated cutaneous	2	Lower limbs	92.12332739	120.332541
8	Lymphocutaneous	5	Lower limbs	49.97764689	145.8153094
9	Disseminated cutaneous	1	Upper limbs	40.27745463	105.6169483
10	Disseminated cutaneous	2	Upper limbs	80.26444335	146.7518219
11	Fixed	5	Face	13.74753296	56.99028894
12	Lymphocutaneous	1	Upper limbs	39.8225295	93.58322097
13	Fixed	2	Upper limbs	43.24949822	75.74668395
14	Fixed	2	Upper limbs	61.11836392	21.56278912
15	Lymphocutaneous	6	Upper limbs	77.17433536	71.32167294

### Statistical Analysis

All results are presented as means ± SEM unless specified otherwise. The two-tailed *t*-test and one-way or two-way ANOVA were used to determine significance between two groups or multiple groups. A *P*-value ≤ 0.05 was considered to reflect statistical significance.

## Results

### Mast Cell-Competent Mice Are More Susceptible to *S. schenckii* Infection Than Mast Cell-Deficient Mice

**Figure 1:**
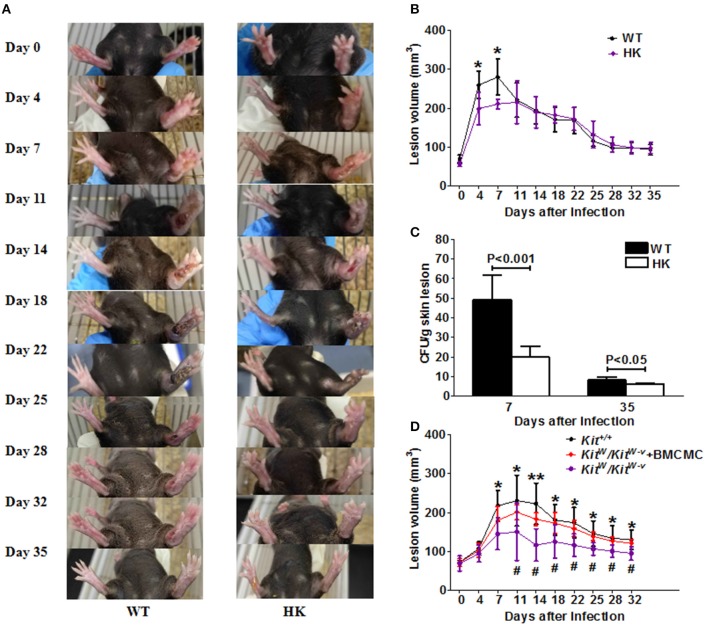
*S. schenkii* infection in MC-deficient mice. **(A–C)** On day 0, 3 × 10^7^ yeasts of *S. schenkii* were injected into the right foot pad of MC-deficient (Hello Kitty, HK, Cpa3-Cre/Mcl-1^fl/fl^) mice and WT littermate control mice. Lesion development was monitored 3-dimensionally over the course of 35 days and calculated as an ellipsoid. **(A)** Representative pictures of *S. schenkii* infection in WT and HK mice during the first 35 days. **(B)** Lesional volume and **(C)** fungal burden of skin lesions were assessed at different time points in infected HK and WT mice. One of 3 experiments with six to eight mice each and similar results is shown. *P < 0.05. **(D)** On day 0, 3 × 10^6^ conidias of *S. schenkii* were injected into the right foot pad of MC-deficient C57BL/6-*Kit*^*W*/^*Kit*^*W*−*v*^ mice and WT (*Kit*^+/+^) littermate control mice. Locally MC-reconstituted *Kit*^*W*/^*Kit*^*W*−*v*^ mice were also used for infections, and the volume of skin lesions was estimated at different time points after infection. Data are expressed as mean ± SEM. *Kit*^+/+^ *vs. Kit*^*W*/^*Kit*^*W*−*v*^, *P < 0.05, **P < 0.01; *Kit*^*W*/^*Kit*^*W*−*v*^ + BMCMC vs. *Kit*^*W*/^*Kit*^*W*−*v*^, ^#^*P* < 0.05, ^*##*^*P* < 0.01. Three independent experiments with six to eight mice per group were performed.

MC-deficient HK (Hello Kitty) mice and WT (Wild type) mice both developed typical skin sporotrichosis lesions with ulceration and scarring after subcutaneous inoculation with *S. schenckii* (Figure 1A). However, WT mice infected with *S. schenckii* developed markedly larger skin lesions compared with HK mice (Figures 1A,B). Differences were most pronounced on day 4 (260 ± 35 vs. 199 ± 42.1 mm^3^, *p* = 0.03) and on day 7 post-infection (280 ± 46 vs. 211 ± 12 mm^3^, *p* = 0.02), with lesion volumes decreased some 25% in HK mice vs. WT mice (Figure 1B). WT mice also exhibited increased lesional fungal burden, more than double as compared to HK mice on day 7 (Figure 1C; 49 ± 13 vs. 20 ± 6 CFU/mm^3^, *p* = 0.002). No inflammation or spores were found in the lungs, spleens, or livers of any of the mice (Supplementary Figure 6A). WT and HK mice showed no spores and similar levels of immune cell infiltration in the draining lymph nodes of sites of infection (Supplementary Figure 6B).

MC-competent WT mice also developed larger sporotrichosis lesions as compared to MC-deficient C57BL/6-*Kit*^*W*^*/Kit*^*W*−*v*^ mice infected with *S. schenckii* (Figure 1D). Reconstitution of skin MCs by local adoptive transfer to C57BL/6-*Kit*^*W*^*/Kit*^*W*−*v*^ mice 4 weeks prior to infection resulted in skin lesions comparable to those of WT mice (Figure 1D). In WT mice, infection with *S. schenckii* resulted in 2-fold increased numbers of MCs at sites of infection, but not non-infected skin sites (Supplementary Figures 1A,B).

### *S. schenckii* Induces MC Cytokine Release, but Not Degranulation

**Figure 2:**
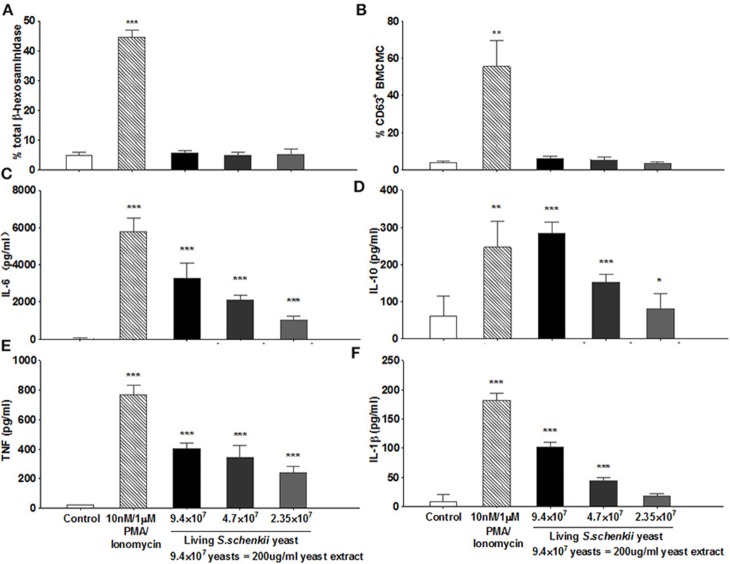
Analysis of MC degranulation and activation following exposure to yeasts from *S. schenckii*. **(A)** β hexosaminidase release by MCs following incubation with supplemented RPMI medium (control), PMA/Ionomycin (10 nM/1 μM), or yeasts of *S. schenckii* for 30 min at 37°C. Values of mediators released from cells incubated with RPMI medium and with PMA/Ionomycin were indicative of spontaneous and degranulation-induced secretion, respectively. *N* = 4. **(B)** WT BMCMCs were incubated with increasing concentrations of *S. schenkii* yeasts, medium alone as negative control, or PMA/Ionomycin as positive control. Expression of CD63 on BMCMCs after 0.5 h of incubation was measured by Flow cytometry, *N* = 6. The release of **(C)** IL-6, **(D)** IL-10, **(E)** TNF, and **(F)** IL-1β into the supernatants after 24 h of incubation was measured by ELISA, N = 3. Data expressed as Mean ± SEM, *P < 0.05, **P < 0.01, ***P < 0.001 were compared with the control group.

**Figure 3:**
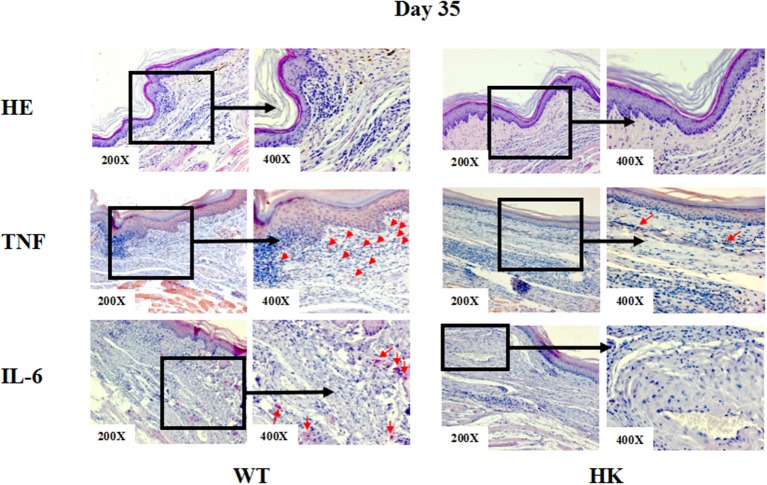
Representative immunohistochemical staining of skin lesions from *S. schenckii* infected WT and HK mice at day 35. Representative immunohistochemical staining of TNF and IL-6 and with HE of skin lesions from *S. schenckii* and *S. albicans-*infected mice at day 35. Red arrows: Positive cells.

**Figure 4:**
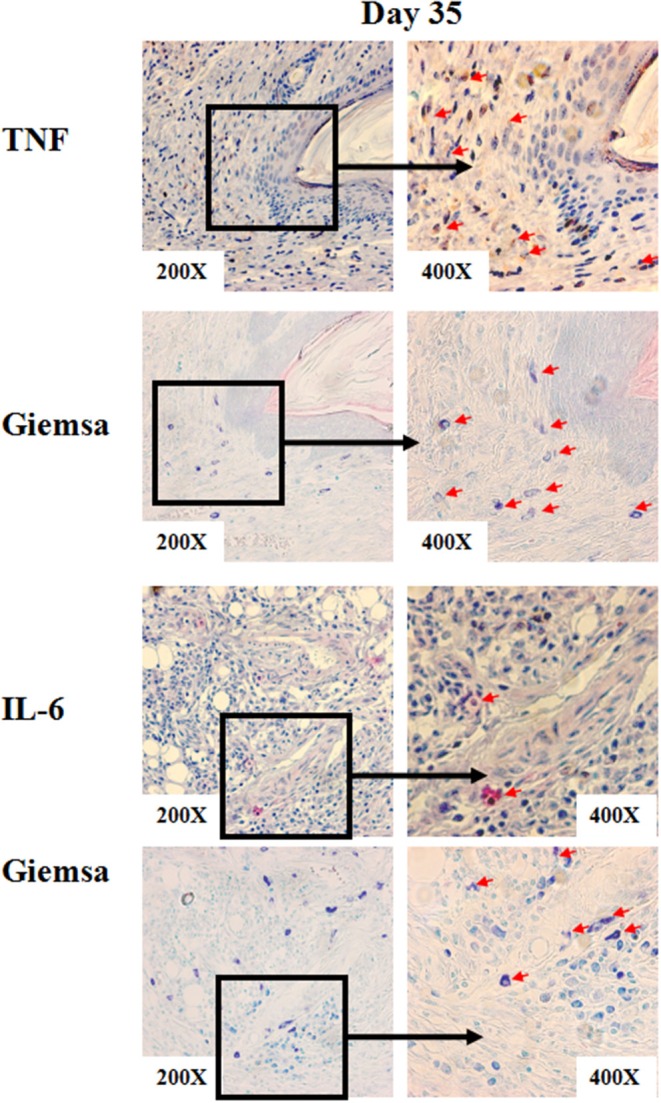
Representative immunohistochemical staining of skin lesions from *S. schenckii*-infected WT mice at day 35. Representative immunohistochemical staining of TNF and IL-6 and with Giemsa of skin lesions from *S.schenckii*-infected WT mice at day 35. Red arrows: Positive cells.

**Figure 5:**
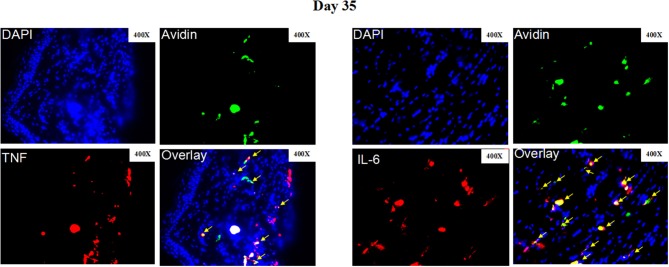
Representative immunofluorescence staining of skin lesions from *S. schenckii*-infected WT mice at day 35. Representative immunofluorescence staining TNF (Red), IL-6 (Red), mast cells (Avidin, green), and DAPI (Blue, nuclei) of skin lesions from *S. schenckii-*infected WT mice at day 35. Red arrows: Double positive cells. Original magnification X40.

To investigate how MCs contribute to *S. schenckii* infections, we tested if MCs degranulate in response to *S. schenckii, in vitro* and *in situ*. MCs did not degranulate *in vitro* as assessed by β-hexosaminidase release and CD63 surface expression by flow cytometry in BMCMCs (Figures 2A,B and Supplementary Figure 2A). Also, MCs did not exhibit signs of degranulation in response to *S. schenckii in situ*, as assessed by quantitative histomorphometric analyses of infected skin sites (Supplementary Figure 1C). In contrast, MCs dose-dependently released substantial amounts of the early response cytokines IL-6, TNF, IL-10, and IL-1β, but not MCP-1, in response to *S. schenckii* (Figures 2C–F and Supplementary Figure 2B). MC-deficient mice showed markedly reduced expression of TNF and IL-6 at sites of *S. schenckii* infection as compared to WT mice (Figure 3). In addition, in WT mice, most cutaneous MCs at sites of *S. schenckii* infection were positive for IL-6 and/or TNF, and most IL-6/TNF positive cells were MCs (Figures 4, 5 and Supplementary Figure 7A).

### Sporothrix Virulence Is Linked to the Induction of MC Cytokine Release

**Figure 6:**
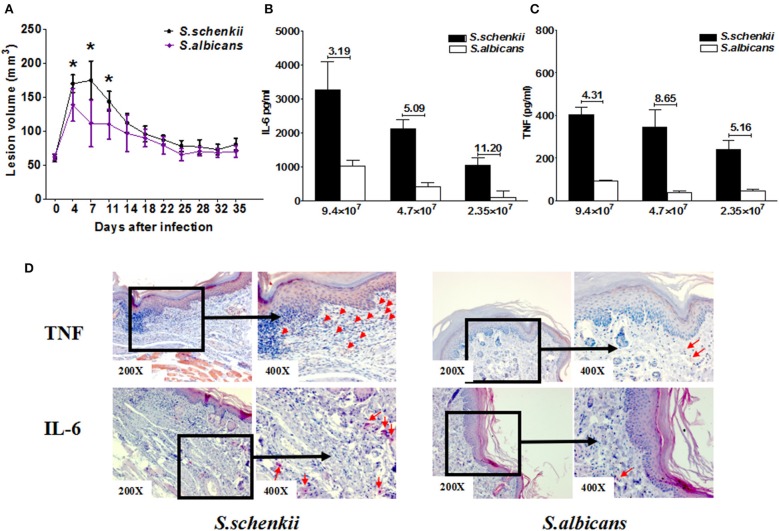
The response of MCs to *S. schenkii* and *S. albicans*. **(A)** *S. schenkii* and *S. albicans* infection in WT mice. 3 × 10^7^ yeasts of *S. schenkii* or *S. albicans* were injected into the right foot pad of WT control mice. Lesion development was monitored 3-dimensionally over the course of 35 days and calculated as an ellipsoid. Data are expressed as mean ± SEM. WT BMCMCs were incubated with increasing concentrations of *S. schenkii* or *S. albicans* yeasts, respectively. Release of **(B)** TNF and **(C)** IL-6 into the supernatants after 24 h of incubation was measured by ELISA, *N* = 3. The numbers on top of the bar mean the fold increase of cytokines induced by *S. schenckii* vs. *S. albicans*. **(D)** Representative immunohistochemical staining TNF and IL-6 of skin lesions from *S. schenckii* and *S. albicans* infected mice at day 35. Red arrows: Staining positive cells. Three independent experiments with six to eight mice per group were performed. *P < 0.05.

To assess the relevance of MC cytokine production in sporotrichosis, we used *S. schenckii* and the less virulent *Sporothrix* species *S. albicans*. We first compared skin responses of mice and then the release of cytokines of MCs exposed to these two *Sporothrix* species. Mice infected with *S. schenckii* developed markedly larger skin lesions than mice infected with *S. albicans* (Figure 6A, 175 ± 38 vs. 112 ± 45 mm^3^ at day 7, *p* = 0.02). In addition, *S. schenckii*-infected mice, but not *S. albicans*-infected mice developed necrosis, with scabbing and ulcerations at sites of infection (Supplementary Figure 3).

When compared for their effects on MC cytokine release, *S. schenkii* induced more than 11-fold and 8-fold higher release of IL-6 and TNF, respectively, as compared to *S. albicans* (Figures 6B,C), whereas IL-10 and IL-1ß release were similar (Supplementary Figures 4A,B). Immunohistochemical analyses showed that TNF and IL-6 were also increased in the skin lesions of *S.schenckii*-infected mice as compared to *S. albicans*-infected mice (Figure 6D and Supplementary Figure 5A).

### TNF and IL-6 Are Increased and Linked to Disease Activity in Patients With Sporotrichosis

**Figure 7:**
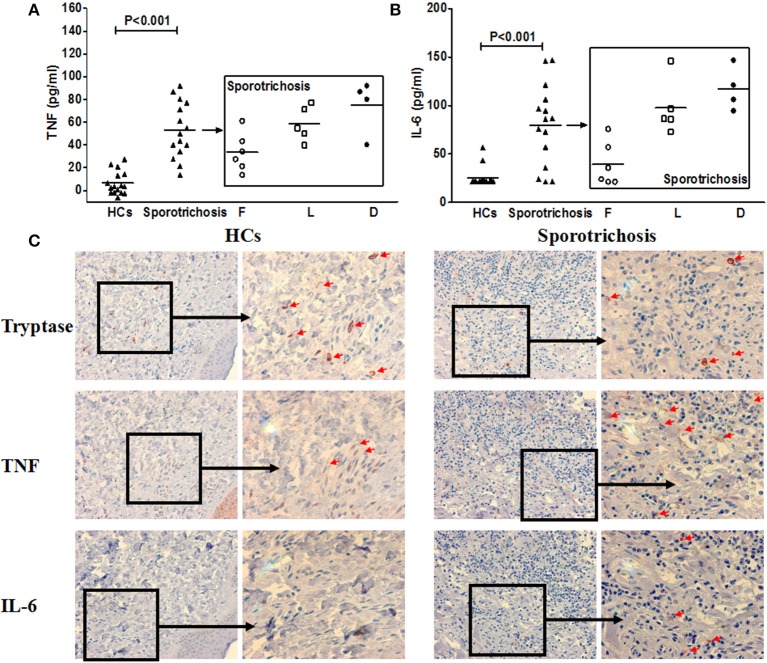
TNF and IL-6 expression in patients with sporotrichosis. Clinical forms of Sporotrichosis: *F*, fixed; *L*, lymphocutaneous; D, disseminated cutaneous. Serum concentrations of TNF **(A)** and IL-6 **(B)** in sporotrichosis patients. **(C)** Representative immunohistochemical staining of tryptase, TNF and IL-6 positive cells in the lesional skin of sporotrichosis patients and healthy control skin.

**Figure 8:**
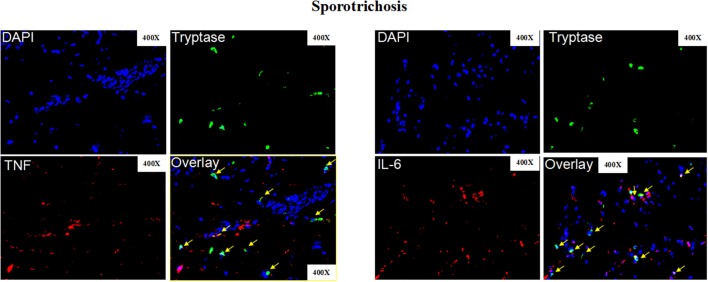
Representative immunofluorescence staining of skin lesions from patients with sporotrichosis. Representative immunofluorescence staining TNF (Red), IL-6 (Red), mast cells (Tryptase, green), and DAPI (Blue, nuclei) of skin lesions from patients with sporotrichosis. Red arrows: Double positive cells. Original magnification X40.

Patients with sporotrichosis showed markedly higher serum levels of TNF and IL-6 than healthy controls (HC; Figure 7; TNF-α: 53 ± 24 vs. 6 ± 10 pg/ml, *P* < 0.001; IL-6: 80 ± 42 vs. 25 ± 10 pg/ml, *P* < 0.001). Serum levels of TNF and IL-6 were highest in patients of disseminated type sporotrichosis, followed by patients with lymphocutaneous sporotrichosis, and lowest in fixed cutaneous sporotrichosis patients (Figures 7A,B). Expression levels of TNF and IL-6 in the lesional skin of sporotrichosis patients were markedly higher as compared to healthy control skin (Figure 7C and Supplementary Figure 5B). Some cutaneous IL-6/TNF-positive cells were MCs, and most MCs were IL-6/TNF-positive (Figures 7C, 8 and Supplementary Figure 7B).

## Discussion

Our study is the first to show that MCs contribute to the pathogenesis of skin sporotrichosis. Mice with normal MC populations that are infected with *S. schenckii* develop larger sporotrichosis lesions than MC-deficient mice. This is, at least in part, due to the production of the proinflammatory cytokines IL-6 and TNF by MCs. MCs showed pronounced production of these cytokines when exposed to *S. schenckii in vitro* or *in vivo*, and the severity of *S. schenckii* infections correlates, in mice and humans with IL-6 and TNF levels.

To investigate the role of MCs in sporotrichosis, we used a modified mouse model with subcutaneous injections of *S. schenckii* into the foot pad of mouse (31, 32). This model shows many features of skin sporotrichosis in patients including ulceration, scarring, and crusty lesions, as well as nodules at infected skin sites (23, 33). By using this model, we found that MC-deficient mice are significantly less susceptible to *S. schenckii* infections: They developed smaller lesions at the site of infection and had lower fungal burden at the peak of infection development. And the differences between MC-deficient mice and WT controls are relatively small at most time points, this may be related to the development process of *S. schenckii* infection. Of importance, our model appears to be well-suited to study acute *S. schenckii* infections of the skin, whereas studies on chronic *Sporothrix* infections, which are common in patients, may require the development of a different model.

Notably, we used two independent MC-deficient mouse models to investigate the role of MCs in sporotrichosis. One was the KIT-independent HK mouse, the other was the Classical KIT-dependent *Kit*^*W*^*/Kit*^*W*−*v*^ mouse. Both models showed very similar results. As *Kit*^*W*^*/Kit*^*W*−*v*^ mice that had been repaired for their skin MC deficiency by adoptive transfer before *S. schenckii* infection showed significantly larger lesions than the non-repaired mice, the effects observed in the *Kit*^*W*^*/Kit*^*W*−*v*^ mouse model appear to be MC-dependent. Our results are supported by an earlier study that showed that the depletion of MCs in mice prior to being subjected to sporotrichosis decreases the fungal load in organs and significantly reduces the severity of infections (19).

Our study does not address through which receptors MCs are activated by *S. schenckii*. MCs have repeatedly been shown to respond to various pathogens through interacting with their toll like receptor (TLR) ligands by secreting cytokines, chemokines, and lipid mediators, while little or no degranulation occurs (34). In addition, fungus-specific IgE-mediated cross linking of the high affinity IgE receptor, Fc_ε_RI, can also result in MC degranulation and the release of preformed mediators as well as the *de novo* synthesis of lipid mediators and cytokines (7). Recently, it has been shown that both yeasts and hyphae of *C. albica* activate MCs through TLR and dectin-1 (12). Thus, MCs may detect *S. schenckii* by TLRs (e.g., TLR-2 and/or TLR-4) or C-type lectin receptors, but further studies are needed to confirm this (35–38).

How do MCs contribute to *S. schenkii* injections? Our findings, together with those of a previous study on peritoneal MCs (22), suggest that the *de novo* synthesis and secretion of proinflammatory cytokines including TNF, IL-6, IL-10, and IL-1β by skin MCs activated by *S. schenckii* promotes skin inflammation, thereby aggravating skin sporotrichosis. This notion is based, in part, on the results of our comparisons of sporotrichosis and MC responses to *S. schenckii* and the less virulent sporothrix species *S. albicans*. First, *S. schenkii* yeasts induced more TNF and IL-6 release from MCs compared with *S. albicans* at the same density. Second, MC-deficient mice showed markedly reduced expression of TNF and IL-6 at sites of *S. schenckii* infection as compared to WT mice. Third, most cutaneous MCs at sites of skin sporotrichosis infection are positive for IL-6 and/or TNF. How exactly do TNF and IL-6 released from MCs make sprorotrichosis worse? The molecular mechanisms remain to be investigated and identified. Importantly, other innate immune cells such as macrophages, neutrophils, and dendritic cells have also been described to release TNF and IL-6 in response to *S. schenckii* infection (32, 39). In addition, both HK and Kit^W^/^Wv^ mice have reduced basophil numbers. Basophils have been shown in several studies to become activated in an IgE-dependent and -independent manner to various fungi (40–42). Therefore, a contribution of basophils cannot be excluded and requires further investigation. The role of basophils in *S. schenckii* infection is still worthy of further investigation.

Are MCs, TNF, and IL-6 involved in human sporotrichosis? The results of our analyses of TNF and IL-6 expression in sporotrichosis patients suggest that this is so. We found the levels of both cytokines to be higher in the serum and skin lesions of affected patients. This is in line with earlier reports that suggest that high levels of TNF, IL-6, and other cytokines can contribute to the pathology of *S. schenckii* infection (43–45). On the other hand, these studies also suggest that very low levels of these cytokines can be harmful in *S. schenckii* infections (43–45). Also, two case reports describe the development of sporotrichosis in patients treated with TNF antagonists (46, 47), whereas there are no reported cases of treatment for sporotrichosis with a TNF antagonist or anti-IL-6. Finally, TNF and IL-6, in the context of sporotrichosis, may also be produced by cells other than MCs, for example T cells and macrophages (43, 48, 49). Taken together, our results suggest, but do not prove, that MC-derived TNF and IL-6 contribute to the pathology of sporotrichosis. The use of drugs that target these cytokines may be promising treatment strategies in patients with sporotrichosis who fail standard therapy.

Our study has several strengths and some limitations. On the plus side, we used two different mouse models of mast cell-deficiency, one KIT-independent, the other KIT-dependent, the latter one complemented by adoptively transferring MCs to MC-deficient recipient mice. Also, we complemented the results from our mouse experiments with human studies. As for limitations, we relied on formalin-fixed skin for our histomorphometric analyses, and not all mast cells, especially in inflamed skin, may be visualized with this fixation. Also, what our current report does not do is provide insights on how MCs are activated by *S. schenckii*, how MCs and the release of cytokines make sporotrichosis worse, and on whether or not MC-targeted treatment or the use of antagonists of MC-derived cytokines can improve the course of sporotrichosis. These questions will have to be answered by ongoing and future studies, including studies that make use of TNF or IL-6 knockout mice or siRNA technology.

Taken together, our present study provides evidence that MCs exacerbate mouse and human skin *S. schenckii* infection and sporotrichosis by releasing TNF and IL-6. Both of these cytokines should be explored for their value as therapeutic targets in the treatment of patients with sporotrichosis. Also, our findings support the notion that the role of MCs in innate immunity to fungal and other pathogens may be more complex than previously thought and that the characterization of MC responses in skin fungal infections merits further investigation.

## Data Availability Statement

All datasets generated for this study are included in the article/Supplementary Material.

## Ethics Statement

The studies involving human participants were reviewed and approved by Department of Dermatology, First Hospital, Peking University, Beijing 100034, China. The patients/participants provided their written informed consent to participate in this study. The animal study was reviewed and approved by Charite - Universitätsmedizin Berlin.

## Author Contributions

Conceptualization and funding acquisition: MM, ZZ, and QJ. Methodology: ZZ, JS, and PG. Software: YL and YW. Validation, formal analysis, and investigation: YL and QJ. Resources: MM, ZZ, JS, and PG. Data curation: QJ, YL, and YW. Writing: QJ. Writing (review and editing): SF, ZZ, RL, and MM. Visualization: QJ, SF, and MM. Supervision: MM, ZZ, and JS. Project administration: MM and ZZ.

### Conflict of Interest

The authors declare that the research was conducted in the absence of any commercial or financial relationships that could be construed as a potential conflict of interest.

## Supplementary Material

Supplementary Figure 1 MCs in skin tissues of mice. Giemsa staining was used to detect MCs in skin tissues of mice. **(A)** Number [mean (S. D.)] of Giemsa-positive cells in skin tissues of left and right foot pad of *S. schenkii* infected WT mice, respectively. **(B)** Representative immunohistological pictures of Giemsa staining in mouse foot pads from *S. schenkii* infected WT mice. **(C)** Skin tissue from the back of WT mice was incubated with increasing concentrations of *S. schenkii* yeasts, medium alone and PMA/Ionomycin as positive control, respectively. Giemsa staining was used to detect the degranulation of MCs in mouse skin tissue. Shown are representative images from three independent experiments.

Supplementary Figure 2 Analysis of CD63 expression and cytokines release of MC in response to yeasts of *S. schenckii*. WT BMCMCs were incubated with increasing concentrations of *S. schenkii* yeasts, medium alone as negative control, or PMA/ionomycin as positive control, respectively. **(A)** Representative flow cytometry analysis of CD63 on BMCMCs after 0.5 h of incubation, *N* = 6. **(B)** Release MCP-1 into the supernatants after 24 h of incubation was measured by ELISA, *N* = 3. ^***^*P* < 0.001 was compared with the control group.

Supplementary Figure 3 *S. schenkii* and *S. albicans* infection in WT mice. 3 × 10^7^ yeasts of *S. schenkii* or *S. albicans* were injected into the right foot pad of WT control mice. Representative pictures of *S. schenkii* or *S. albicans* infection in WT mice during the 35 days.

Supplementary Figure 4 The response of MCs to *S. schenkii* and *S. albicans*. WT BMCMCs were incubated with increasing concentrations of *S. schenkii* or *S. albicans*, respectively. Release of **(A)** IL-1β and **(B)** IL-10 into the supernatants after 24 h of incubation was measured by ELISA, *N* = 3. The numbers on top of the bar mean the fold increase of cytokines induced by *S. schenckii* vs. *S. albicans*.

Supplementary Figure 5 TNF and IL-6 expression in skin tissues. **(A)** Average of TNF or IL-6 positive stained area percentage in skin tissues of right foot pad of *S. schenkii* or *S. albicans* infected WT mice, respectively. **(B)** Average of Tryptase, TNF or IL-6 positive stained area percentage in the lesional skin of sporotrichosis patients and healthy control skin, respectively.

Supplementary Figure 6 Representative HE staining of internal organs in mice with *S. schenkii* infection. **(A)** Representative immunohistological pictures of HE staining in mice from *S. schenkii* infected WT and HK mice. **(B)** Representative HE staining pictures from skin tissues of WT mice with injection of *S. schenkii* yeasts. Shown are representative images from three independent experiments.

Supplementary Figure 7 Co-localization of mast cells and IL-6 or TNF expression in skin tissues. **(A)** Average percentage of IL-6 or TNF positive stained cells on mast cells (Avidin positive) or Avidin positive stained cells (Mast cells) on IL-6 or TNF positive stained cells in skin tissues of right foot pad of *S. schenkii* infected WT mice at day 35, respectively. **(B)** Average percentage of IL-6 or TNF positive stained cells on mast cells (Tryptase positive) or Tryptase positive stained cells (Mast cells) on IL-6 or TNF positive stained cells in the lesional skin of sporotrichosis patients, respectively.

Supplementary Figure 8 Number of MCs in skin tissues of mice. Mast cell was counted in randomly selected fields (X200) of baseline control after 4 wk in Kit^W^/Kit^W−v^ reconstituted with wild type MCs, There is no difference in the mast cell number in skin of WT mice and Kit^W^/Kit^W−v^ mice reconstituted with wild type mast cells 4 wk after adoptive transfer. Three independent experiments with 6 mice per group were performed for each experiment.
