# Supplementary figures and images for: Skin Mast Cells Contribute to Sporothrix schenckii Infection
Source: Front Immunol. 2020 Mar 19;11:469. doi: 10.3389/fimmu.2020.00469 (PMC7096480; doi:10.3389/fimmu.2020.00469)

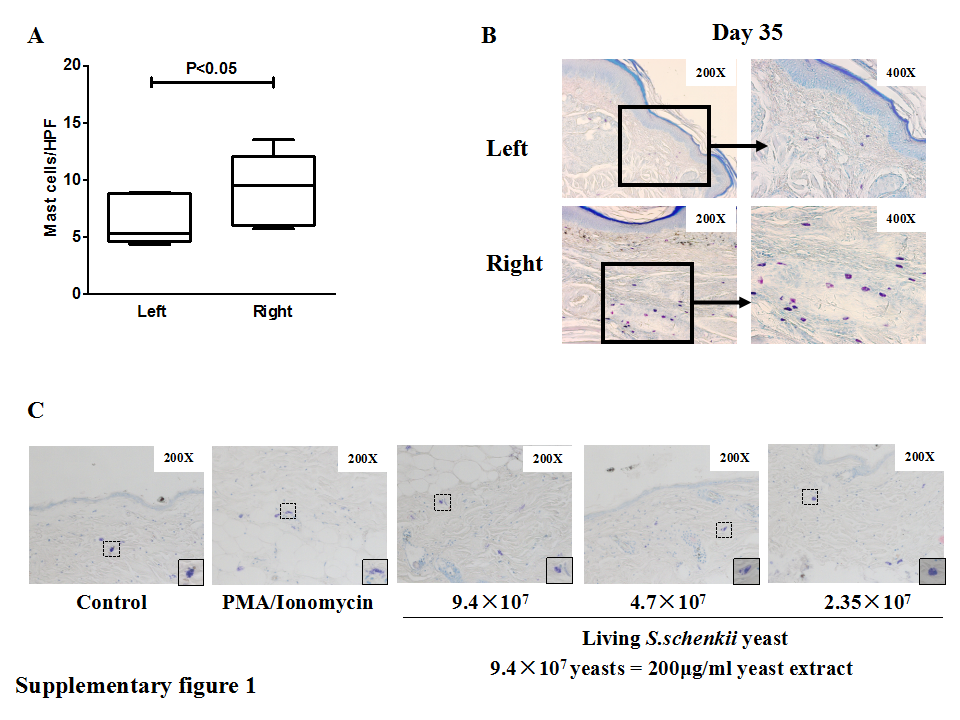

Supplement: Supplementary Figure 1 — MCs in skin tissues of mice. Giemsa staining was used to detect MCs in skin tissues of mice. (A) Number [mean (S. D.)] of Giemsa-positive cells in skin tissues of left and right foot pad of S. schenkii infected WT mice, respectively. (B) Representative immunohistological pictures of Giemsa staining in mouse foot pads from S. schenkii infected WT mice. (C) Skin tissue from the back of WT mice was incubated with increasing concentrations of S. schenkii yeasts, medium alone and PMA/Ionomycin as positive control, respectively. Giemsa staining was used to detect the degranulation of MCs in mouse skin tissue. Shown are representative images from three independent experiments. [file Image_1.TIF]

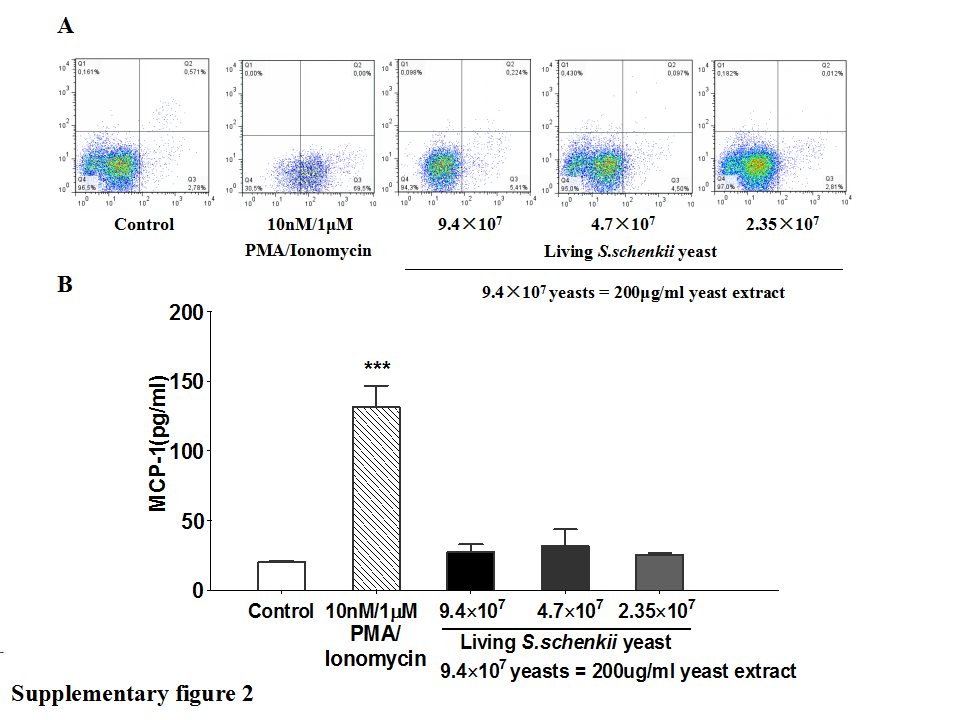

Supplement: Supplementary Figure 2 — Analysis of CD63 expression and cytokines release of MC in response to yeasts of S. schenckii. WT BMCMCs were incubated with increasing concentrations of S. schenkii yeasts, medium alone as negative control, or PMA/ionomycin as positive control, respectively. (A) Representative flow cytometry analysis of CD63 on BMCMCs after 0.5 h of incubation, N = 6. (B) Release MCP-1 into the supernatants after 24 h of incubation was measured by ELISA, N = 3. ***P < 0.001 was compared with the control group. [file Image_2.TIF]

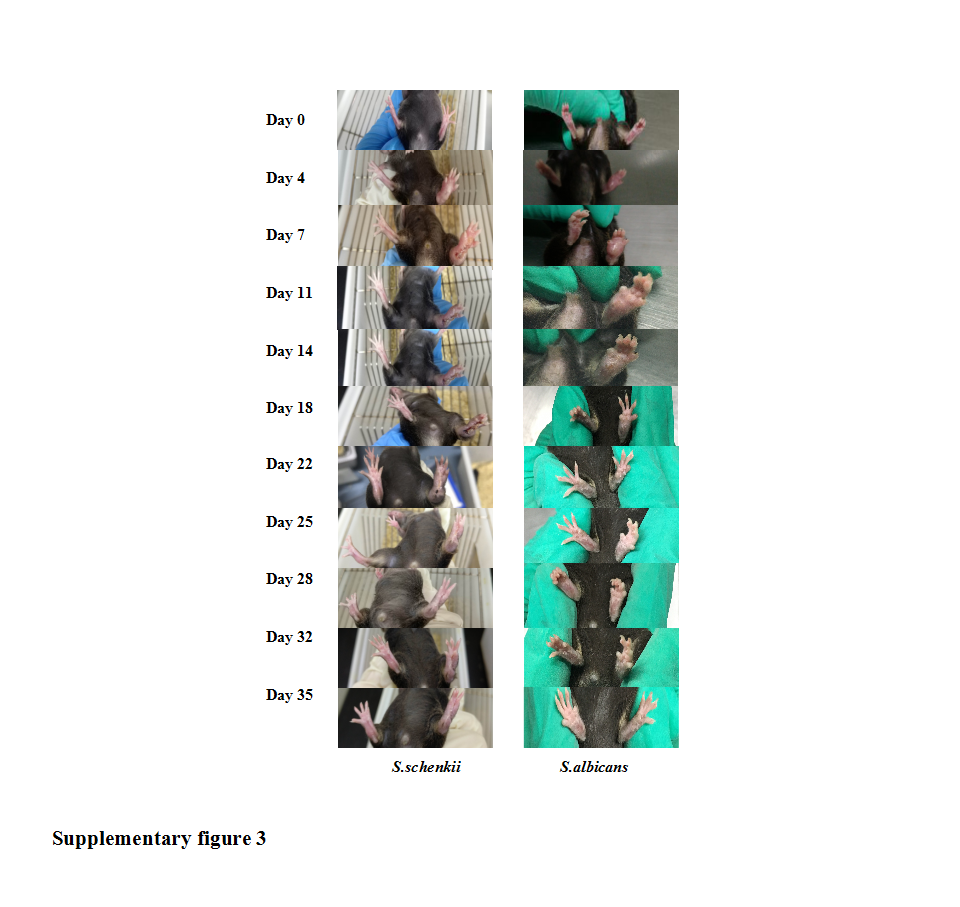

Supplement: Supplementary Figure 3 — S. schenkii and S. albicans infection in WT mice. 3 × 10^7^ yeasts of S. schenkii or S. albicans were injected into the right foot pad of WT control mice. Representative pictures of S. schenkii or S. albicans infection in WT mice during the 35 days. [file Image_3.TIF]

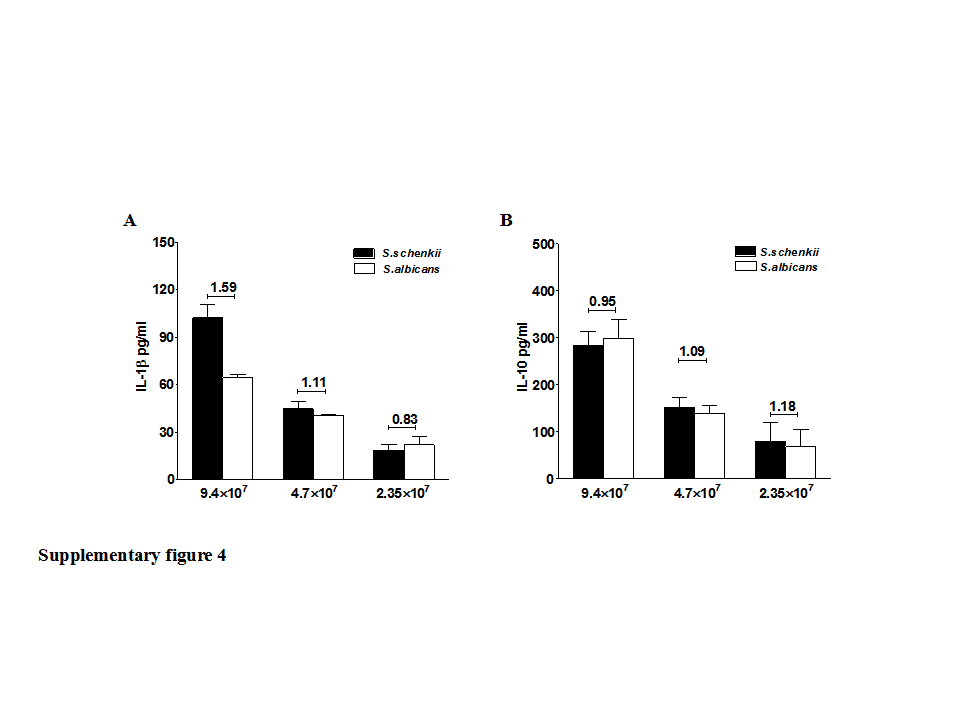

Supplement: Supplementary Figure 4 — The response of MCs to S. schenkii and S. albicans. WT BMCMCs were incubated with increasing concentrations of S. schenkii or S. albicans, respectively. Release of (A) IL-1β and (B) IL-10 into the supernatants after 24 h of incubation was measured by ELISA, N = 3. The numbers on top of the bar mean the fold increase of cytokines induced by S. schenckii vs. S. albicans. [file Image_4.TIF]

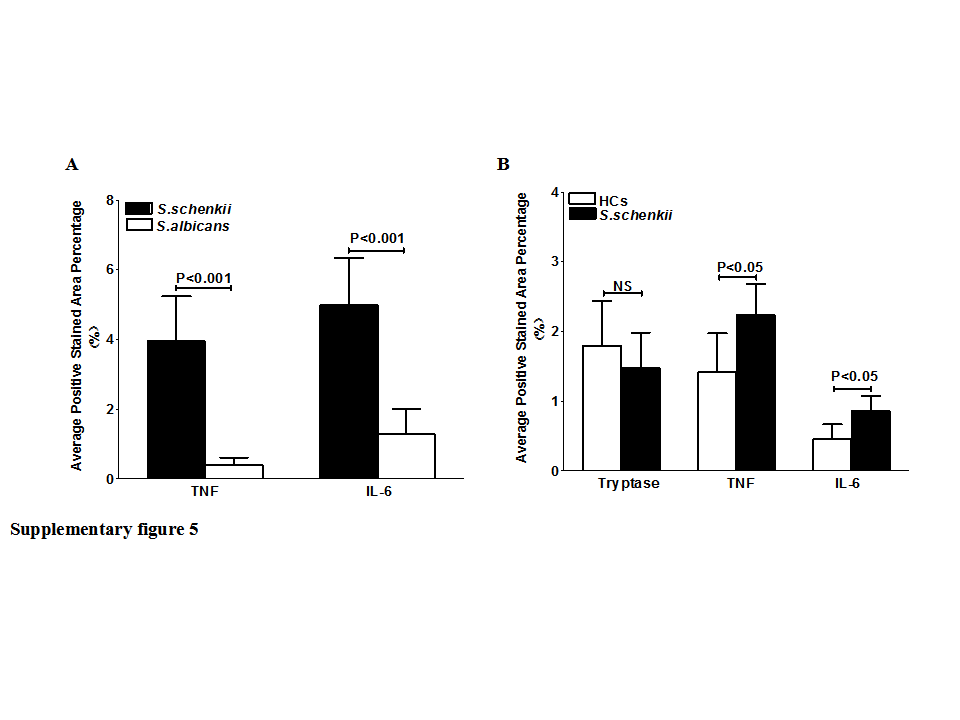

Supplement: Supplementary Figure 5 — TNF and IL-6 expression in skin tissues. (A) Average of TNF or IL-6 positive stained area percentage in skin tissues of right foot pad of S. schenkii or S. albicans infected WT mice, respectively. (B) Average of Tryptase, TNF or IL-6 positive stained area percentage in the lesional skin of sporotrichosis patients and healthy control skin, respectively. [file Image_5.TIF]

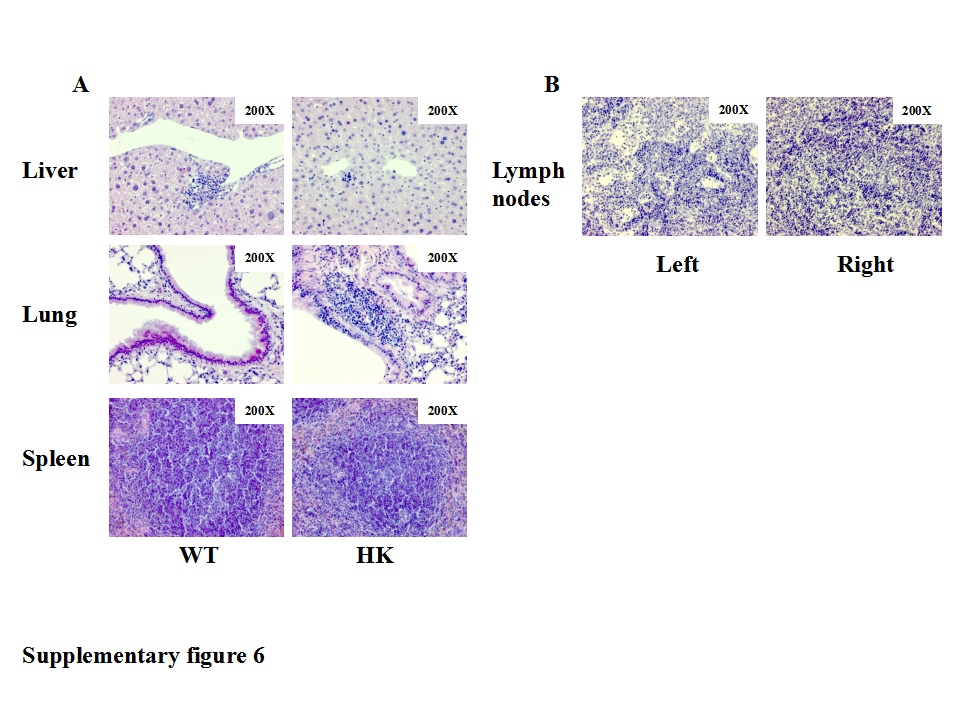

Supplement: Supplementary Figure 6 — Representative HE staining of internal organs in mice with S. schenkii infection. (A) Representative immunohistological pictures of HE staining in mice from S. schenkii infected WT and HK mice. (B) Representative HE staining pictures from skin tissues of WT mice with injection of S. schenkii yeasts. Shown are representative images from three independent experiments. [file Image_6.TIF]

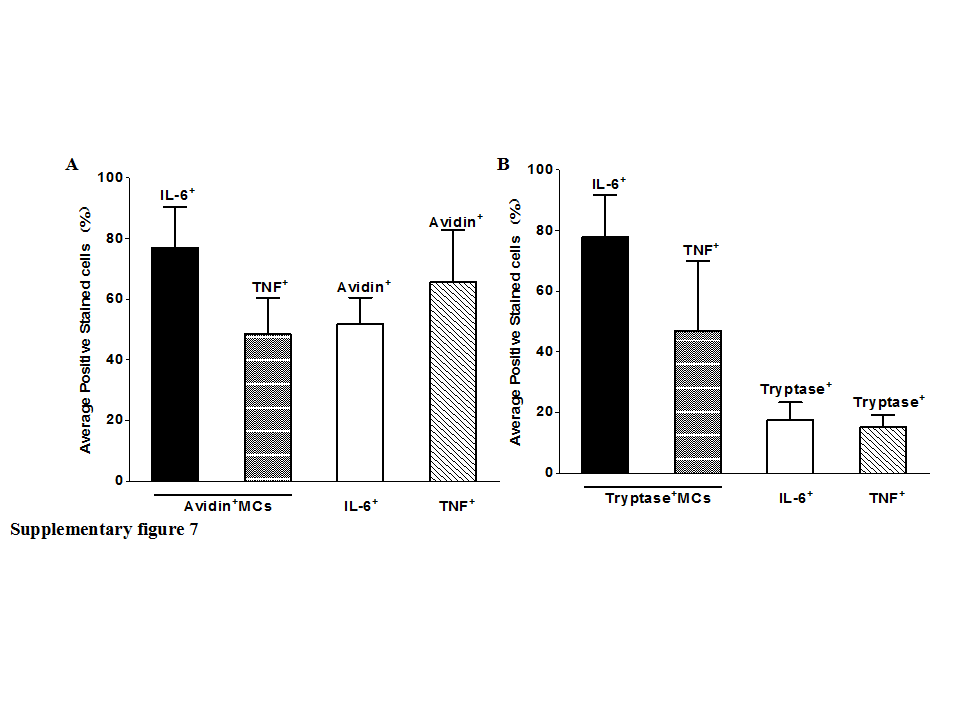

Supplement: Supplementary Figure 7 — Co-localization of mast cells and IL-6 or TNF expression in skin tissues. (A) Average percentage of IL-6 or TNF positive stained cells on mast cells (Avidin positive) or Avidin positive stained cells (Mast cells) on IL-6 or TNF positive stained cells in skin tissues of right foot pad of S. schenkii infected WT mice at day 35, respectively. (B) Average percentage of IL-6 or TNF positive stained cells on mast cells (Tryptase positive) or Tryptase positive stained cells (Mast cells) on IL-6 or TNF positive stained cells in the lesional skin of sporotrichosis patients, respectively. [file Image_7.TIF]

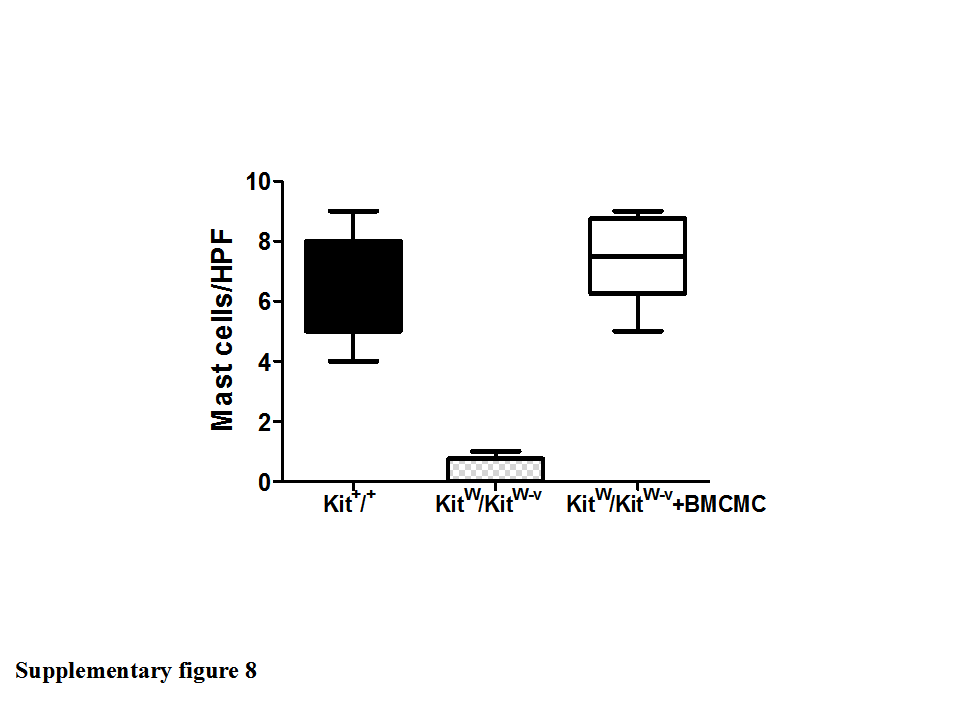

Supplement: Supplementary Figure 8 — Number of MCs in skin tissues of mice. Mast cell was counted in randomly selected fields (X200) of baseline control after 4 wk in KitW/KitW−v reconstituted with wild type MCs, There is no difference in the mast cell number in skin of WT mice and KitW/KitW−v mice reconstituted with wild type mast cells 4 wk after adoptive transfer. Three independent experiments with 6 mice per group were performed for each experiment. [file Image_8.TIF]
